# Reporting adverse events of COVID-19 vaccines: The case of Bulgaria

**DOI:** 10.1371/journal.pone.0269727

**Published:** 2022-06-10

**Authors:** Vanya Rangelova, Ralitsa Raycheva, Sara Sariyan, Ani Kevorkyan

**Affiliations:** 1 Department of Epidemiology and Disaster Medicine, Faculty of Public Health, Medical University of Plovdiv, Plovdiv, Bulgaria; 2 Department of Social Medicine and Public Health, Faculty of Public Health, Medical University of Plovdiv, Plovdiv, Bulgaria; 3 Faculty of Medicine, Medical University of Plovdiv, Plovdiv, Bulgaria; University of Hail, SAUDI ARABIA

## Abstract

As a member state of the European Union, where vaccines against COVID-19 are available and affordable, Bulgaria reports the lowest immunization coverage and the most pronounced vaccine distrust. The present study aimed to assess the self-reported adverse reactions following COVID-19 vaccination as a possible tool to increase the trust in vaccines. A cross-sectional survey-based study, covering 761 vaccinated respondents, was conducted in Plovdiv (469 with an mRNA vaccine and 292 with an adenoviral vector vaccine). Descriptive statistics parametric and non-parametric methods were applied. Statistical significance was set at p<0.05. The median age of the respondents was 42 years, females (72.5%). At least one adverse reaction was reported in 89.9% of those immunized with mRNA vaccine and 93.8% in the adenoviral vector vaccine group (p>0.05). They were mild to moderate and resolved within several days. The levels of local reactions were comparable: 91.7% in those who received mRNA and 89.7% in those who received an adenoviral vector vaccine (p = 0.366). The most common types of systemic reactions were fatigue, headache, and muscle pains. An association was found between the systemic reactions and the type of vaccine administered: 59.7% in mRNA recipients and 89.4% in adenoviral vector vaccinees (p<0.001). None of the registered systemic reactions required medical attention. There were 3 reports of generalized urticaria after an mRNA and 2 after an adenoviral vector vaccine. The reported reactions are relatively high but expected and no adverse events have been reported that are not listed in the official Summary of Product Characteristics.

## 1. Introduction

Historically, vaccines have been widely recognized as a key tool for the successful prevention and control of communicable diseases. The emergence of a new, pandemic pathogen—SARS-CoV-2- at the end of 2019 [1] put humanity in need of urgent action through the worldwide integration of intellectual and financial resources. Both pharmaceutical and non-pharmaceutical countermeasures have been used to deal with this global threat. Immunization strategies with available vaccines against COVID-19 provide the greatest hope and most effective way to achieve herd protection [2]. On the other hand, many unresolved issues, such as the duration of post-vaccination protection, the need for additional doses, the emergence and circulation of new variants, and the fear of potential short- or long-term side effects are some of the reasons for COVID-19 vaccine hesitancy [3, 4].

Among the countries in the European Union (EU) / European Economic Area (EEA), where vaccines against COVID-19 are available and affordable, Bulgaria reports the lowest immunization coverage [5] and the most pronounced vaccine distrust [6, 7]. According to the COVID-19 Unified Information Portal, as of December 13, 2021, a total of 3 494 341 doses of vaccines against COVID-19 have been delivered in Bulgaria, or 31.8% of the population (over 18 years) have completed a vaccination course. This indicator is two and a half times lower than the one registered in the EU / EEA–the cumulative uptake of full vaccination among adults (18+) is 78.6% (according to ECDC COVID-19 Vaccine Tracker). The 14-day notification rate of COVID-19 deaths reflects this low use of vaccination, with deaths in our country many times higher than the average for the EU/EEA. At the end of week 48 in 2021, the 14-day COVID-19 death rate in Bulgaria was 233.8 per million population (for the EU / EEA was 55.9 deaths) [8].

While in some parts of the world (mainly in sub-Saharan Africa) inequalities in access to vaccines have emerged as a major problem [9], in others, vaccine hesitancy is a major barrier to controlling the spread of the virus. In 2019, the World Health Organization set out hesitancy regarding immunizations and the associated risks to society’s health in the list of the ten biggest threats to human health worldwide [10]. Trust in vaccines must also be complemented by trust in the institutions responsible for vaccination. Lack of acceptance of vaccination may derive from previous failures of health systems and public institutions to serve certain population groups effectively and engender their trust. In general, trust in institutions is critical for the effective functioning of society and acceptance of the public policy, particularly so during a crisis [11]. Gathering reliable information on the level of post-vaccine adverse events and making it publicly available might increase public confidence in vaccines and immunization strategies at the national level, as well as in health policies in the country.

The aim of the present study was to assess the self-reported adverse reactions following COVID-19 vaccination as possible complementary information to the overall measures and tools to increase the immunization coverage and trust in vaccines.

## 2. Materials and methods

### 2.1. Settings and sample size

In the period March to September 2021, a cross-sectional study using a semi-structured questionnaire was conducted in the city of Plovdiv, Bulgaria. A non-random convenience sampling method was used in recruiting participants from two out of four immunization centers in the town of Plovdiv. The potential respondents were asked to provide their emails if willing to participate in the survey during their visit/s for the administration of a COVID-19 vaccine. In addition, the snowball method was applied to sample individuals who received their doses as part of the national campaign to prevent targeted populations at-risk–healthcare workers and university staff members–started at the beginning of 2021 (UMHAT “St. George”, UMHAT “Palmed”, UMHAT “Kaspela”, Medical University Plovdiv, etc.). The sample size was calculated using G*Power version 3.1.9.7 [12]. The maximum amount by which the sample results may differ from the full population (margin of error) and the probability that the sample accurately reflects the attitudes of the targeted population (confidence interval) were set to 5% and 95%, respectively. Based on similar studies, the expected frequency (outcome probability) is assumed to be 60% as the prevalence of side effects following COVID-19 vaccines ranged between 62% to 93% [13–15]. Thus, the number of people who need to take the survey was estimated to be 383. The total sample size achieved included 761 respondents, vaccinated with one of the available vaccines against COVID-19.

### 2.2. Participants and eligibility criteria

1) age ≥18 years; 2) voluntary participation without financial compensation and opportunity to withdraw at any moment until data submission; 3) vaccination with any of the available vaccines, regardless of previous COVID-19 infection.

### 2.3. On-line questionnaire

The self-administered questionnaire, split into four sections, was prepared using the Microsoft Forms Platform (Microsoft Forms. Microsoft 365 Package) and disseminated via email through the electronically generated link. The questionnaire’s front page included an informed consent form with an opt-out option if the respondents were not willing to continue.

The first section gathered information about demographics, professional occupation, comorbidities of the participants, and information on previous COVID-19 infections and adverse events following immunization in the past.

The second section collected information about the first dose of the COVID-19 vaccine, i.e. type of vaccine administered: mRNA vaccine–Comirnaty (BNT162b2 (INN: tozinameran) by Pfizer-Biontech), Spikevax (mRNA-1273 (INN: elasomeran) by Moderna), adenoviral vector vaccine–Vaxzevria (INN: ChAdOx1-S [recombinant]) by Astra-Zeneca), COVID-19 Vaccine Janssen (INN: Ad26.COV2-S [recombinant] by Johnson & Johnson); the occurrence of adverse events following immunization–local reactions (pain, edema, redness, rash, nodule, infected abscess, sterile abscess, cellulitis), systemic reactions (chills, fatigue, headache, muscle pains, joint pains, nausea/vomiting, diarrhoea, reduced appetite, lymphadenopathy), allergic reactions (anaphylaxis, angioedema, generalized urticaria) [16], and reactions of interest with regard to COVID-19 vaccines (thrombocytopenia, blood disorders, syncope, arthralgia). Moreover, concerning the local and systemic reactions, the participants were asked to define the severity of the reaction (mild, moderate, or severe) and the time point of occurrence (24 hours after vaccination, 24–48 hours after vaccination, more than 72 hours after vaccination).

The third section’s questions were identical to the previous one, with a focus on the second dose of the COVID-19 vaccine.

In section four, the participants had the opportunity to provide additional information not included in the questionnaire but considered important (about reactions or complaints they had after receiving the vaccine).

In the analysis of the data, without detailing the manufacturer of the administered vaccine, we considered the levels of adverse events following vaccination (local and systemic) in two groups—immunized with mRNA or adenoviral vector vaccine.

### 2.4. Survey response rate

Overall, 975 questionnaires were sent by email to people vaccinated with one of the available vaccines against COVID-19, and 761 responses were collected which yielded a 78% response rate.

### 2.5. Statistical analysis

Standard descriptive statistics was used to summarize demographic characteristics. Quantitative variables were presented by the mean and standard deviation (mean ± SD) or median (25th percentile; 75th percentile), based on the sample distribution. Qualitative variables were presented as numbers absolute/relative frequencies, totals, and percentages (n, %). The Kolmogorov-Smirnov test was applied to inform about the distribution of the patients sampled. Differences between observed and theoretical distributions were tested using the chi-square test for independence. Differences between proportions were tested using the z-test. The non-parametric Friedman test was applied to test for differences between groups when the dependent variable being measured is ordinal. The paired data regarding the post-vaccine adverse events after the first and second vaccination was analyzed by using Wilcoxon signed-rank test. Statistical significance was set at p<0.05. Statistical analyses were performed using SPSS Statistics v. 26 software (IBM Corp. Released 2019. Armonk, NY: USA).

### 2.6. Ethics statement

The study received an ethical exemption from the Ethics Committee as it met one of the criteria for exemption (an anonymous survey or interview that does not involve the collection of identifiable data). The study complies with the principles of the Declaration of Helsinki for medical research involving human subjects [17].

## 3. Results

There were 716 responses for the first dose (n = 351 Comirnaty, n = 76 Spikevax, n = 286 Vaxzevria, n = 3 COVID-19 Vaccine Janssen) and 653 for the second dose of the vaccine, respectively (n = 358 Comirnaty, n = 73 Spikevax, n = 222 Vaxzevria).

### 3.1. Demographics

The median age of the respondents was 42 years (25th percentile 32 yrs.; 75th percentile 52 yrs.), predominantly female (n = 552; 72.5%) (Table 1). Most of the participants were under 50 years of age (n = 547, 71.9%). The most common comorbidities reported were arterial hypertension (n = 61, 29.3%), disorders of the thyroid gland (n = 54, 25.9%), and diabetes (n = 17, 7.8%).

**Table 1:** Demographic characteristics of the respondents.

Variables	n (%)
**Gender**	
**Male**	206 (27.1)
**Female**	552 (72.5)
**Other**	3 (0.4)
**Healthcare personnel**	218 (28.6)
**Comorbidities**	
**Yes**	208 (27.3)
**No**	553 (72.7)
**Have you been infected with COVID-19?**	
**Yes**	117 (15.4)
**No**	653 (84.6)

The total number of people immunized with an mRNA vaccine was 469, of which 422 (89.9%) reported at least one adverse event, and of the 292 immunized with a vector vaccine, 274 (93.8%) reported an adverse event (Pearson χ 2 test = 0.082, p> 0.05). In general, most reported adverse events were mild to moderate and resolved within several days.

### 3.2. Local reactions after vaccination

Post-vaccine reactions, when dose number was not considered, were reported by 91.7% (430/469) in those who received mRNA and 89.7% (262/292) in those who received an adenoviral vector vaccine (Pearson χ^2^ test = 0.837, p = 0.366). When examining the reported local reactions after the first dose for the mRNA and the adenoviral vector vaccines we were not able to prove an association between the type of vaccine administered and the occurrence of a local reaction. The most reported local reaction after receiving the first dose of both mRNA and adenoviral vector vaccines was pain at the injection spot: 90.7% (n = 388) for mRNA vaccines and 88.2% (n = 255) for adenoviral vector vaccines. Most of the respondents graded the pain as mild (61.6% for mRNA vaccines vs. 55.7% for adenoviral vector vaccines) and reported the time point of manifestation within the first 24 hours after receiving the vaccine (85.1% for mRNA vaccines and 78.8% for adenoviral vector vaccines).

Concerning the local reactions following the administration of the second dose of a vaccine, we were able to demonstrate a statistically significant difference between the type of vaccine and the frequency of occurrence of a local reaction after the second dose administration. A significantly higher proportion of respondents who received the mRNA vaccine reported pain (87.0%) and edema (22.0%) at the injection spot. Most of the respondents graded the pain as mild (74.7% for mRNA vaccines vs. 86.2% for adenoviral vector vaccines) and that it occurred in the first 24 hours after administration of the vaccine (84.0% for mRNA vaccine recipients vs. 86.8% for adenoviral vector vaccine recipients) (Table 2).

**Table 2:** Local reactions after first and second dose with mRNA and vector vaccines.

Variables	1st dose (n = 716)		p-value	2nd dose (n = 653)		p-value
	mRNA n = 427	Vector n = 289		mRNA n = 431	Vector n = 222	
**Pain, n (%)**	388 (90.7)	255 (88.2)	0.260	375 (87.0)	152 (68.5)	**0.000**
**Edema, n (%)**	110 (25.8)	70 (24.2)	0.662	95 (22.0)	25 (11.3)	**0.001**
**Redness, n (%)**	97 (22.7)	65 (22.5)	1.000	73 (16.9)	33 (14.9)	0.576
**Rash, n (%)**	23(5.4)	8 (2.8)	0.096	15 (3.5)	3 (1.4)	0.136
**Nodule, n (%)**	51 (12.0)	28 (9.7)	0.395	38 (8.8)	13 (5.9)	0.219
**Infected abscess, n(%)**	7 (1.6)	2 (0.7)	0.325	2 (0.5)	0	0.551
**Sterile abscess, n (%)**	6 (1.4)	1 (0.3)	0.251	2 (0.5)	0	0.551
**Cellulitis, n (%)**	7 (1.6)	2 (0.7)	0.325	4 (0.9)	0	0.305
**Local reactions, n (%)**	393(92.0)	258 (89.3)	0.233	377 (87.5)	155 (69.8)	**0.000**

### 3.3. Systemic reactions after vaccination

In general, the most common types of reaction were fatigue, headache, and muscle pains. When comparing systemic reactions, an associative relationship was found with the type of vaccine administered: 59.7% (280/469) in mRNA vaccine recipients vs. 89.4% (261/292) in adenoviral vector vaccine recipients (Pearson χ^2^ test = 71.15; p<0.001). Systemic reactions after the first dose of vaccine occurred more frequently after adenoviral vector vaccines compared to mRNA vaccines (Table 3). After the second dose of the vaccine, systemic reactions were more common after mRNA vaccines compared to adenoviral vector vaccines. None of the registered systemic reactions required medical attention.

**Table 3:** Systemic reactions after first and second dose with mRNA and adenoviral vector vaccines.

Variables	1st dose (n = 716)		p-value	2nd dose (n = 653)		p-value
	mRNA n = 427	Vector n = 289		mRNA n = 431	Vector n = 222	
**Chills, n (%)**	107 (25.1)	223 (77.2)	**0.000**	180 (41.8)	63 (28.4)	**0.001**
**Elevated temperature, n (%)**	81 (19.0)	205 (70.9)	**0.000**	156 (36.2)	58 (26.1)	**0.011**
**Fatigue, n (%)**	193 (45.2)	221 (76.5)	**0.000**	233 (54.1)	94 (42.3)	**0.005**
**Headache, n (%)**	126 (29.5)	181 (62.6)	**0.000**	144 (33.4)	72 (32.4)	0.861
**Muscle pains, n (%)**	112 (26.2)	184 (63.7)	**0.000**	167 (38.7)	53 (23.9)	**0.000**
**Joint pains, n (%)**	79 (18.5)	127 (43.9)	**0.000**	112 (26.0)	41 (18.5)	**0.032**
**Nausea/Vomiting, n (%)**	30 (7.0)	43 (14.9)	**0.001**	37 (8.6)	11 (5.0)	0.113
**Diarrhea, n (%)**	10 (2.3)	14 (4.8)	0.089	15 (3.5)	8 (3.6)	1.000
**Reduced appetite, n (%)**	37 (8.7)	88 (30.4)	**0.000**	57 (13.2)	22 (9.9)	0.255
**Lymphadenopathy, n (%)**	39 (9.1)	20 (6.9)	0.333	41 (9.5)	5 (2.3)	**0.000**
**Systemic reactions, n (%)**	248 (58.1)	260 (89.9)	**0.000**	287 (66.6)	110 (49.5)	**0.000**

### 3.4. Allergic reactions after vaccination

Another type of event that may occur following immunization is allergic reactions. There were 3 reports of generalized urticaria after the second dose of an mRNA vaccine, and 2 after the second dose of a vector vaccine, respectively.

### 3.5. Adverse events following vaccination depending on the age

For this purpose, we distributed the participants into two groups–people under or above 50 years of age. There is an association between the age of the respondents under 50 years of age and the occurrence of adverse events following immunization (Table 4.).

**Table 4:** Adverse events following vaccination depending on the age after 1st and 2nd dose, respectively.

Variables	1st dose (n = 716)		p-value	2nd dose (n = 653)		p-value
	<50 y.o. (n = 525)	≥50 y.o (n = 191)		<50 y.o. (n = 460)	≥ 50 y.o. (n = 193)	
**Local reactions, n (%)**	492 (93.7)	159 (83.2)	**0.000**	378 (82.2)	154 (79.8)	0.508
**Elevated temperature, n (%)**	239 (45.5)	47 (24.6)	**0.000**	173 (37.6)	41 (21.2)	**0.000**
**Systemic reactions, n (%)**	393 (74.9)	115 (60.2)	**0.000**	294 (63.9)	103 (53.4)	**0.014**

## 4. Discussion

In the history of vaccine development and regulation, concern has focused on both vaccine efficacy (and correlates of clinical protection) and vaccine safety. Both vaccine efficacy and vaccine safety are relative: no vaccine is 100 per cent efficacious or 100 per cent safe [18]. There are currently four vaccines available in the European Union (EU) to prevent COVID-19: mRNA-based vaccines (Comirnaty, Pfizer-BioNTech and Spikevax, Moderna) and adenoviral vector vaccines (Vaxzevria, AstraZeneca, and COVID-19 Janssen Vaccine), after obtaining authorization for conditional marketing authorization use from the European Medicines Agency (EMA). Chronologically, Comirnaty received the first such permit on 21 December 2020, followed by Spikevax on 6 January 2021, Vaxzevria on 29 January 2021, and COVID-19 Janssen Vaccine on 11 March 2021. These authorizations are based on quality data, safety, and efficacy of the medical product, accumulated worldwide. Bulgaria, as part of the EU, has a full range of vaccines, and since the end of December 2021 a total of 8,248,670 doses of vaccines have been delivered (Comirnaty– 4,365,270; Janssen– 1,777,300; Spikevax– 1,183,200 and Vaxzevria– 1,183,200). With a population of 6,878,470 in the country, the successful administration of the vaccine would lead to a much higher immunization coverage than the reported 31.8% of the population (over 18 years) (at the end of week 49 of 2021), defined as the lowest in Europe (ECDC COVID-19 Vaccine Tracker). Reasons for this low coverage are complex and include distrust of the government, and the authorities, and last (but not least) scepticism and distrust in the safety of vaccines by certain parts of Bulgarian society. Some of the distrust might be due to the very short period between the declaration of a pandemic in March of 2020 by the WHO, and the availability of the first vaccines in December 2020.

Plovdiv is the second-largest city in Bulgaria and the total population of the region was estimated to be 666 398 people at the end of 2020 [19]. Approximately 200,000 citizens were vaccinated at the end of 2021, which is 30.0% coverage and the ratio of mRNA vs. adenoviral vector vaccine uptake is 2:1. We were able to recruit 761 respondents: 469 were immunized with an mRNA vaccine, of which 422 (89.9%) reported an adverse reaction vs. 292 immunized with an adenoviral vector vaccine, of which 274 (93.8%) reported such. Similar high levels of adverse reactions have been reported in other studies: in Saudi Arabia, (85.6% of BNT162b2 vaccinees and 96.05% of ChAdOx1 vaccinees) [20] in Afghanistan (93.5% when immunized with ChAdOx1 vaccine (AstraZeneca)) [21], in Poland (93.9% after the first dose of Pfizer and 96.5% after Astra Zeneca) [22] slightly lower values were reported from Ecuador after the first dose of 79.0% and the second dose of 75.1% of the BNT162b2 vaccine [23].

The levels of local post-vaccine reactions without taking into account the dose sequence were high: 91.7% (430/469) in those who received mRNA and 89.7% (262/292) in those who received an adenoviral vector vaccine. Comparative research by the US Centers for Disease Control (CDC) in people vaccinated with Pfizer and receiving a placebo reported at least one local reaction in the vaccinated group in 84.7% [24]. Local reactions regardless of the type of vaccine administered are the most common in many studies related to the reactogenicity of the applied vaccines against COVID-19 [20–27]. Our results demonstrated that pain at the injection spot was the most common local reaction, and this finding is consistent with findings from other studies regarding the mRNA vaccines [14, 24] and a study from Ramasamy et al. concerning the administration of the AstraZeneca vaccine [28].

When comparing systemic reactions, a relationship was found with the type of vaccine administered. One of the most common types of reactions was fatigue and the finding is consistent with several other studies [14, 24, 28, 29].

The frequency of reporting of adverse events following immunization was more common in younger respondents and this reflects the results reported by other authors [14, 30]. In a study from the UK, individuals below 55 years of age had significantly higher levels of side effects following immunization with both mRNA and viral vector vaccines [30]. Younger adults may report more adverse events probably due to their robust immune system, than older adults [30, 31].

Since the beginning of the vaccination campaign in Bulgaria to the end of November 2021, the total number of reports submitted to the Bulgarian Medicines Agency (BDA) for post-vaccination reactions after administration of vaccines against Covid-19 is 3,696, with 3,201,859 doses of vaccines. This represents 0.1% (Spikevax—359; Comirnaty—908; Vaxzevria—2 286; and Janssen– 143). Our data are based on self-reporting; no additional medical monitoring was performed. This confirms that the adverse events after vaccination were mild to moderate in severity and did not require hospitalization.

A Gallup study in 2018 before the surge of SARS-CoV-2 showed significant differences in attitudes towards vaccines in different regions of the world [32]. In high-income regions, 72% of people in North America and 73% in Northern Europe agree that vaccines are safe. In Western Europe, this figure is lower (59%) and in Eastern Europe, it is only 50%. In low-income regions, trust in vaccines is exceptional—95% of people in South Asia and 92% in East Africa.

Thus, this preliminary negative attitude among the countries in Eastern Europe, including Bulgaria, deepened further against the background of the short period for the appearance of the COVID-19 vaccines. According to a survey conducted by Eurobarometer in 2021, Bulgarians are the most sceptical about coronavirus vaccines of all nations in the world, with 26% saying they will never be vaccinated, which puts us in first place in the EU [33]. This probably applies to the medical staff in the country, where only 27.8% have completed a vaccination course.

The COVID-19 pandemic has highlighted how a lack of clear information and timely data can cause uncertainty in decision-making and foster mistrust in the population [11]. Two systematic reviews that investigate the factors which might affect the general vaccine uptake and the seasonal influenza vaccine discovered a strong association between the vaccine uptake and the perception of vaccine safety and adverse events following immunization [34, 35]. The same concern has been reported about the safety of COVID-19 vaccines in two similar studies [3, 36]. However, the participants in one of the studies [3] who were unsure about accepting the COVID-19 vaccine stated that they might choose to receive the vaccine if they received credible information about the vaccine’s safety and effectiveness.

Adverse events because of vaccination are not rare and are proof that the immune system is responding [22]. Ongoing surveillance for the potential emergence of adverse effects is also essential to support public trust, using well-developed pharmacovigilance systems to track problems or adverse reactions not detected in the clinical trials. Reducing uncertainty associated with the vaccine will always encourage vaccination. Thus, exploring adverse events following immunization and presenting the results to the general public might be helpful as an informative tool to increase trust in COVID-19 vaccines and acceptance. Harrison et al. [37] suggest that to increase public trust in vaccines we need to communicate better the risks and benefits of vaccines. One of the possible ways to achieve this goal is to provide meaningful, evidence-based information on the known risks of vaccination [38] such as the vaccine adverse events databases.

The universe of vaccine information, science, safety research, quality control, and policy decisions is large and complex. Both professionals and the public poorly understand many aspects of the system. Pertinent information needs to be communicated in a strategic and comprehensive manner to reach the overarching goal of informed decision-making [18]. However, the success of vaccination campaigns will largely be influenced by the extent to which people trust the effectiveness and safety of the vaccines, the competence, and reliability of the institutions that deliver them, and the principles that guide government decisions and actions [11].

## 5. Conclusion

Proportions of vaccinated people reporting adverse events found by us in both mRNA and adenoviral vector vaccines are high, but these adverse events are mild and self-limiting, similar to those reported in different parts of the world. Reported adverse events are expected according to the summary of product characteristics of the vaccines administered, and no adverse events have been reported that are not listed in the approved vaccine information.

### Limitations and strengths

The study has several limitations. First, this is a self-reported adverse events online survey without clinical follow-up and medically confirmed relations by healthcare professionals and it may impact result reporting due to differences in interpretation and tolerance thresholds from patient to patient. The data are self-reported, and can reflect reporting biases, such as under- or over-reporting of adverse events or their severity. Further, although the requirement for the sample size was reached there is still a potential for reporting bias, based on the self-reported nature of the questionnaire as well as self-selection bias due to the voluntary participation in the survey. Nevertheless, despite the listed limitations this is the first study on the topic conducted in the Bulgarian setting and could initiate further research.
